# Gangliosides in Podocyte Biology and Disease

**DOI:** 10.3390/ijms21249645

**Published:** 2020-12-17

**Authors:** Berkan Savas, Giuseppe Astarita, Massimo Aureli, Dil Sahali, Mario Ollero

**Affiliations:** 1 INSERM, IMRB, Univ Paris Est Créteil, F-94010 Créteil, France; berkan.savas@inserm.fr (B.S.); dil.sahali@inserm.fr (D.S.); 2 Department of Biochemistry and Molecular & Cellular Biology, Georgetown University, 20007 Washington, DC, USA; gastarita@gmail.com; 3 Department of Medical Biotechnology and Translational Medicine, University of Milano, Milano Italy, 20090 Segrate (Milano), Italy; massimo.aureli@unimi.it; 4 Service Néphrologie, AP-HP, Hôpital Henri Mondor, F-94010 Créteil, France

**Keywords:** glycosphingolipids, ceramide, podocytopathies, glomerulopathies, glomerulus, kidney, rafts, nephrotic syndrome

## Abstract

Gangliosides constitute a subgroup of glycosphingolipids characterized by the presence of sialic acid residues in their structure. As constituents of cellular membranes, in particular of raft microdomains, they exert multiple functions, some of them capital in cell homeostasis. Their presence in cells is tightly regulated by a balanced expression and function of the enzymes responsible for their biosynthesis, ganglioside synthases, and their degradation, glycosidases. The dysregulation of their abundance results in rare and common diseases. In this review, we make a point on the relevance of gangliosides and some of their metabolic precursors, such as ceramides, in the function of podocytes, the main cellular component of the glomerular filtration barrier, as well as their implications in podocytopathies. The results presented in this review suggest the pertinence of clinical lipidomic studies targeting these metabolites.

## 1. An Overview of Sphingolipid and Glycosphingolipid Metabolism

**Figure 1:**
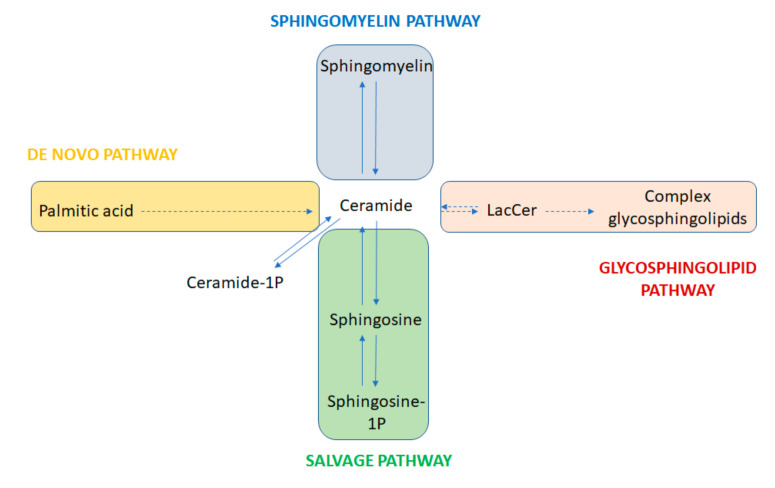
Global view of the sphingolipid metabolism. Simplified representation of the four main pathways encompassing the synthesis of sphingolipid species, with ceramides as central compounds. Plain arrows indicate single reactions. Dashed arrows denote multiple reactions. De novo and sphingomyelin pathways result in ceramide synthesis from palmitic acid and sphingomyelin as ultimate precursors, respectively. The so-called salvage or catabolic pathway results in the production of the bioactive sphingosine-1 phosphate. Ceramide itself can be phosphorylated into the bioactive ceramide-1 phosphate. Finally, the hydrolytic or glycosphingolipid pathway leads to ceramide glycation. Galactosyl-ceramide is the precursor of sulfatides, not shown in the figure. The rest of the complex glycosphingolipids (lactosides, globosides, cerebrosides, and gangliosides) are derived from lactosyl ceramide (LacCer). All pathways are reversible except the de novo synthesis.

Sphingolipids represent one of the eight categories that the Lipid MAPS consortium currently classifies as lipids. They are characterized by the presence of a sphingoid base backbone, consisting of an aliphatic long chain amino alcohol, most frequently represented by sphingosine. Sphingolipids constitute a highly heterogeneous category of molecules, with a central group of compounds, ceramides, containing a fatty acid moiety associated with the sphingoid base via an amide bond [1]. The high diversity of sphingolipids is the result of four different anabolic and catabolic pathways that converge and diverge to and from the ceramide backbone (Figure 1). These are known as the “de novo” synthesis, the hydrolytic, the sphingomyelin, and the catabolic or salvage pathways.

**Figure 2:**
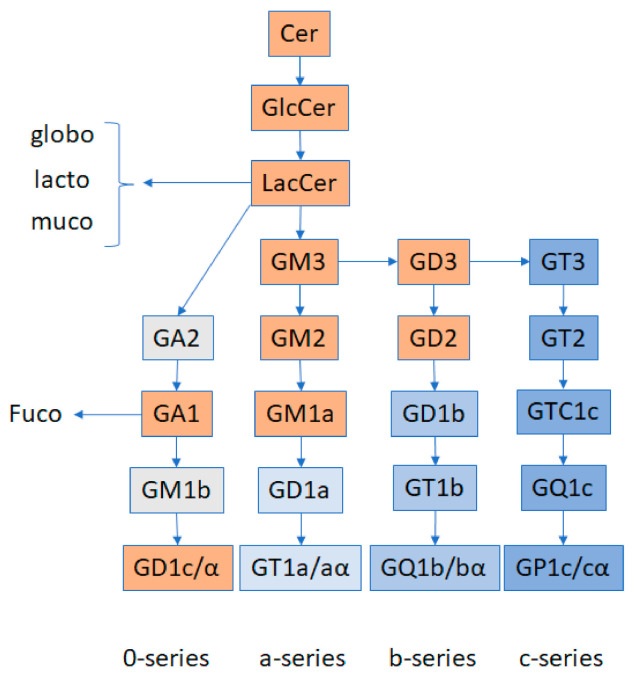
Schematic representation of ganglioside biosynthesis pathways. LacCer are the precursors of the globo, lacto, muco, and ganglio series of glycosphingolipids. GM3 are the precursors of the a, b, and c series of gangliosides. LacCer are precursors of asialo (0-series) gangliosides (GA). GA1 give way to fucosylated glycosphingolipids. “/” denotes two different structures of gangliosides produced from the same precursor. “α” denotes specific ganglioside structures in which one sialic acid residue is branched to N-acetylgalactosamine (GalNAc). All the other sialic acid residues are branched to galactose residues (modified from [5]). The orange colored rectangles denote the molecular species whose abundance has been reported to date as changed in the context of podocytopathies (summarized in Table 1). Many of these reactions are reversible. GM: Monosialo gangliosides; GD: Disialo gangliosides; GT: Trisialo gangliosides; GQ: Quadrisialo gangliosides; GP: Pentasialo gangliosides.

Ceramides are highly versatile compounds that can undergo glycosylation, among other modifications. Glycosylation of ceramides through the so-called hydrolitic pathway generates the glycosphingolipid category, encompassing several hundred compounds. Ceramide glycosylation leads to glucosylceramide (GlcCer), then through galactosylation to lactosylceramide (LacCer). LacCer is the ultimate precursor of complex glycosphingolipids, namely globosides, cerebrosides, and gangliosides. Within glycosphingolipids, gangliosides are defined by the presence, with some exceptions, of at least one sialic acid residue, a derivative of neuraminic acid, associated with an oligosaccharide chain. A 5-N-acetyl derivative of neuraminic acid is the most abundant form of sialic acid in humans, with 10% corresponding to the 5-N-acetyl-9-O-acetyl derivative [2]. Three main pathways produce all the gangliosides (Figure 2). Monosyalilated ganglioside M3 (GM3) is the simplest molecule in the pathway, and the precursor of all gangliosides of the a-, b-, and c- series. It contains one single sialic acid residue and is produced by sialylation of LacCer by the GM3 synthase (ST3GAL5) [3]. An excellent description of the ganglioside structure, structural variability, and their implications for interaction with other membrane molecules is provided in the publication by Mauri and Colls [1].

Gangliosides are particularly abundant in cellular membranes, due to the hydrophobicity of their ceramide backbone. Their presence is highly related to the membrane function and associated with the cell homeostasis, as they are among the most abundant and characteristic components of the specialized microdomains defined as lipid rafts. Their hydrophilic heads are much bulkier than those of glycerophospholipids; this feature determines their lateral separation properties, resulting in the aggregation and generation of a positive curvature [1].

Lipid rafts or liquid-ordered microdomains are very rich in lipids containing saturated or little unsaturated acyl chains in their hydrophobic moieties [4]. Ceramides and their glycated derivatives, gangliosides, correspond to this description, and consequently they are some of the main lipid components of rafts. Therefore, the ganglioside function in the cell is associated with their chemical structure, lateral mobility, and their ability to partition in raft-like environments.

As key membrane components, gangliosides are involved in the regulation of cell signaling, intercellular crosstalk, pathogen invasion, apoptosis/survival, proliferation/differentiation, and immune cell function. Such a diverse array of functions implies a tight regulation of their anabolism and catabolism, whose dysfunction results in a number of pathologies.

## 2. Podocytes: A Complex Structure and a Singular Membrane Organization

Podocytes are highly specialized visceral epithelial cells constituting the glomerular filtration barrier in the kidney, along with endothelial cells and the basement membrane. They ensure the impermeability of the barrier to high molecular weight molecules, most of the blood proteins, by an intricate system of interdigitated cellular prolongations (foot processes) and a particular organization of cell junctions (slit diaphragm). Alterations in these structures lead to increased permeability, resulting in proteinuria. This is a characteristic feature of podocytopathies, which includes IgA nephropathy, membranous nephropathy, and idiopathic nephrotic syndrome (INS). Podocytopathies can also be secondary to other morbidities, such as diabetes (diabetic nephropathy) and systemic lupus erythematosus (lupus nephritis).

Some individual features of the lipid composition of podocytes have been known for a long time, contributing to a progressive comprehension. Though a global view of the podocyte lipidome is still lacking, despite the information provided by a few lipidomic studies [6,7,8]. Cholesterol was early revealed as a key component of podocyte membrane. Initially found in normal rat podocytes as especially present in the urinary side and less abundantly in the basal side [9], it was shown as a key component of the slit diaphragm through its interaction with podocin [10], one of the resident proteins in this raft-like structure [11].

Another slit-diaphragm protein, nephrin, was found associated with lipid rafts, and this association was necessary for nephrin phosphorylation, a modification related to slit diaphragm integrity [12]. Its presence in rafts, defined as TritonX-100 insoluble microdomains, was found partial, meaning that only one pool of cellular nephrin is associated with these structures. That pool was found to interact with podocin and the actin cytoskeleton via the CD2-associated protein (CD2AP) adaptor [13,14]. Mutations in nephrin and podocin genes, which are at the origin of genetic nephrotic syndrome, are known to abolish lipid raft localization [15,16], highlighting the relevance of these microdomains to podocyte function. Lipid rafts in the podocyte have also been proven involved in the regulation of transient receptor potential canonical type 6 (TRPC6) channels [17], which play a key role in the regulation of podocyte morphology and the pathogenesis of INS. The ensemble of these reports indicate that podocyte lipid rafts and the slit diaphragm correspond to very close structures and display a tight functional association, with raft resident proteins contributing to the slit diaphragm spatial organization [18]. The role of sphingolipids in the podocyte has been extensively studied and reviewed by Fornoni and Colls [19].

## 3. Gangliosides in Podocytes

Despite the extreme structural diversity of gangliosides, only a handful of them have been identified in podocytes and their functions are outlined in physiologic and pathologic conditions. A pioneering biochemical and immunohistochemical work mapped several gangliosides in different segments of kidneys from different species, also comparing the developing and the mature organ [20]. In the glomerulus, a transition from more complex to less complex gangliosides was observed along the organ maturation. A global view of the ganglioside content within the sphingolipid landscape in podocytes has been provided by comprehensive profiling on immortalized human podocytes by a combination of high performance thin layer chromatography (HPTLC), radioactive labeling of sphingosine, and mass spectrometry [8]. A significant majority of the identified sphingolipids correspond to neutral forms, encompassing sphingomyelin, ceramide, GlcCer, LacCer, globotrihexosylceramide, ganglioteraosylceramide, and globopentaosylceramide. GM3 accounted for 5% of total podocyte sphingolipids, while GM1b and GD1α were also identified. This can be considered as a reference sphingolipid profiling of human podocytes. Nevertheless, immortalized cells must always be considered as a model, in which molecular expression and distribution could differ from the in vivo conditions. Moreover, the sphingolipid profile of human cells can differ from that of animal models, as seen below.

### 3.1. O-Acetylated GD3

Interrogations on the biological relevance of podocyte gangliosides emanate from three independent studies. The pioneering work detecting a ganglioside as a main component of podocytes consisted of the identification of the antigen recognized by a monoclonal antibody specific of glomerular podocytes in rat kidney [21]. This antigen was characterized by ion exchange, thin layer and gas-liquid chromatography as O-acetylated-GD3. Subsequently, the same antibody was used to isolate podocyte rafts [12]. Acetylation of the sialic acid moiety can have profound consequences in ganglioside function. Another monoclonal antibody had been previously found to stain the renal cortex and the recognized antigen characterized as a glycolipid migrating between GM2 and GM1 in thin layer chromatograms [22]. From this point, O-acetylated GD3 has become a marker of podocytes and used to characterize and validate podocyte cell lines [23]. GD3 O-acetylation has been related to viral infection and to the resistance to apoptosis of tumor cells [24]. Moreover, 9-O-acetyl-GD3 has been found specifically increased in rat kidney in response to lead exposure, which produces microalbuminuria [25].

Injection of puromycin aminonucleoside in rats induces a glomerulopathy known as puromycin aminonucleoside nephrosis (PAN). This is considered as an in vivo model of the human INS form minimal change nephrotic syndrome (MCNS). The group that developed the monoclonal antibody to detect O-acetylated GD3 studied its expression in the kidney of proteinuric PAN rats. They found a significant decrease of this antigen and its precursor GD3, suggesting impaired GD3 synthase (ST8Sia1) and O-acetyltransferase activities. These changes were observed to precede the onset of proteinuria [26]. Consistent with these results, the RNA levels of GD3 synthase are decreased in the adriamycin injection model of focal and segmental glomerulosclerosis (FSGS, one of the INS forms) in mice [27]. Neither GD3 nor O-acetylated GD3 have been identified in human podocytes [8], which indirectly challenges both the relevance of rodent models and that of immortalized cell models in reproducing disease features. The results of PAN stimulation are in any case extremely important to underline the implication of gangliosides in the regulation of podocyte function, and point at O-acetylation as a modification that could profoundly impact ganglioside function.

### 3.2. GM3 and GD3

To date, one of the most relevant and comprehensive functional studies on podocyte gangliosides was an elegant work by Jin and Colls [6]. The beauty of their finding is the fact that gangliosides were indirectly identified as extremely important in podocyte biology. They used immunoprecipitation to find the interactors of the soluble vascular endothelial growth factor (VEGF) receptor 1 (Flt1) on the surface of podocytes, resulting in the unbiased detection by mass spectrometry of gangliosides, and further GM3, as the main interactor. Additional functional studies linked this interaction with cytoskeleton reorganization, which is a cornerstone in the function of the glomerular barrier.

Immunohistochemical and electron microscopy analyses of human kidney specimens have confirmed the specific presence of GM3 in the foot processes of podocytes [28], comforting the functional data described in the previous paragraph [6]. This confirms the high abundance of this type of ganglioside in podocytes, and its participation in maintaining the functional integrity of the filtration barrier. As a negatively charged molecule, it contributes to the protein-impermeability of the structure. This implies that any alterations in the metabolic pathways leading to decreases in GM3 might result in altered foot processes and in permeability to proteins.

Another unbiased study was performed by our team on mouse podocytes transfected with a vector encoding the protein CMIP (c-maf inducing protein), a podocyte and lymphocyte marker of INS [29]. Untargeted differential lipidomics of CMIP-expressing and non-expressing cells resulted, surprisingly, in a downregulation of the most abundant gangliosides (GM3, GM2, and GD2), while GM3 was identified as the most abundant podocyte component of the ganglio series. Interestingly, GM1 was not decreased [7]. The results suggested that an alteration in the pathway leading to ganglioside biosynthesis could play a role in the pathogenesis of INS. Once more, it places GM3 as a main actor in podocyte and glomerular function. However, in a previous study on the mouse Adriamycin model of nephrotic syndrome, the RNA levels of GM3 synthase were unchanged [27].

It has been proposed that GM3 regulates by direct interaction both the insulin and the epidermal growth factor (EGF) receptors [30,31,32,33]. In diabetes-associated nephropathy (DN), glomerular hypertrophy and proteinuria are observed. While the levels of circulating gangliosides have been correlated with albuminuria in DN patients [34], GM3 has been found to increase in the kidney and other tissues, along with glucocerebroside, in a streptozotocin-induced model of diabetes [35,36]. Therefore, GM3 appears as an actor of podocyte injury in DN. However, in another study on the same diabetic rat model, several gangliosides, mainly GM3, were found to decrease in glomeruli, in parallel to a reduction in sialic acid content [37]. A more recent work compared the tubular and glomerular content in GM3 in rat models of type 1 and type 2 diabetes. GM3 was found to increase in renal tubules in both models as compared to controls, but glomeruli showed a weak increase in type 1 and no change in type 2 diabetes [38]. In all these works, GM3 was semi-quantified by thin layer chromatography and its tissue localization determined by immunofluorescence with a monoclonal antibody. Using mass spectrometry imaging, glomerular and tubular ganglioside levels have been reported as increased, along with other lipid classes, in a DN mouse model [39]. The development of lipidomics should provide a final answer to the intriguing dynamics of the GM3 profile associated with diabetes [3,30].

GM3 levels can be regulated either by ST3GAL5 or by the degrading enzyme neuraminidase 3 (NEU3), or by both. In the form of nephritis developed by more than half of the patients with lupus erythematosus, an increased presence of hexosylceramides and LacCer (the ganglioside immediate precursors), and a decreased NEU sialidase activity have been reported in the kidney and in urine [40]. Although the increased NEU activity should be associated with decreased GM3 levels, the latter were unexpectedly increased in a mouse model of lupus nephritis [41], suggesting a complex imbalance between the synthesis and catabolism of gangliosides.

A question inferred from these studies is whether the experimental blocking of ganglioside biosynthesis impacts renal function. In this line, *ST3GAL5* knockout mice have been developed and have provided capital information. For instance, insulin signaling is enhanced upon GM3 synthase invalidation [42]. However, to date no renal phenotype has been described in this model. A podocyte specific inducible model of *ST3GAL5* invalidation could help complete the picture of GM3 function in glomeruli.

Interestingly, alpha-galactosidase A activity has been found significantly decreased in blood specimens from FSGS patients in hemodialysis as compared to non-FSGS control patients [43]. This enzyme is mutated in Fabry disease, in which lysosomal accumulation of globotriaosylceramide has been reported. LacCer is also the precursor of the lacto and globo series (Figure 2), and an impaired flux to GM3 and other gangliosides could be associated, as a cause or a consequence, with a detoured metabolism to globosides in FSGS. The data available to date are insufficient to establish a concrete hypothesis and a thorough analysis of these pathways in FSGS should be performed.

### 3.3. GM2

Mutations in the genes encoding the enzymes involved in glycosphingolipid metabolism result in lysosome storage disorders [44], characterized by the accumulation of specific lipids depending on the metabolic reaction blocked by the mutation. For example, Sandhoff disease, due to hexosaminidase deficiency, leads to accumulation of GM2, since hexosaminidase is responsible for the removal of N-acetylgalactosamine (GalNAc) from GM2 and subsequent retroconversion to GM3. This was first detected in the brain and hepatic tissues from a patient, where lipid analysis of the kidney revealed an accumulation of globoside [45]. In Fabry disease, accumulation of globotriaosylceramide leads to proteinuria and podocyte injury [46].

Mesangial cells, another cell component of glomeruli, undergo hypertrophy and proliferation in DN. This is induced in vivo by glucosamine administration, which results in increased levels of GM1 and GM2. Exogenous administration of these two molecules also leads to the same effects and points to GM2 accumulation by hexosaminidase deficiency as a mechanism of glomerular alteration in DN [47].

### 3.4. GA1

LacCer is not only the precursor of GM3 and, subsequently of a- and b-series gangliosides, but also a particular class of glycosphingolipids known as asialo-gangliosides or 0-series gangliosides (Figure 2). This includes GA2 and GA1, respectively containing one and two galactose residues in addition to glucose and GalNAc, but exceptionally no sialic acid. In the context of DN, an untargeted, unbiased metabolomic study has recently pointed at GA1 as a circulating marker of renal function [48]. In the study, diabetic patients where subdivided into three groups according to their estimated glomerular filtration rate (eGFR), whose low levels are characteristic of renal dysfunction. The results identified plasma levels of GA1 as negatively correlated with eGFR, suggesting the former as a precocious marker of kidney damage and risk of end stage renal disease. This is to our knowledge the only work showing a ganglioside as a circulating biomarker of renal disease. It is difficult to associate this finding with the above described alterations in the ganglioside profile in diabetic glomeruli. It could be a sign of either a block in alpha-2,3-sialyltransferase (ST3GAL1), which would be accompanied by accumulation of GM1a, GD1b, GT1c, or by increased desialylation of GM1b due to NEU1/4 activity. It could also be a sign of increased flux towards ganglioside synthesis.

## 4. Lessons from APOL1 Genetic Variants

A polymorphism in the gene encoding apolipoprotein L1 (APOL1) has been associated with the development of several nephropathies, including hypertension-attributed nephropathy, and glomerulopathies like HIV-associated nephropathy (HIVAN) and idiopathic FSGS. The pathogenic mechanism linking the variant *APOL1* alleles with podocyte damage concerns the lysosomal and mitochondrial functions, as well as the autophagic flux [49,50]. An exhaustive study inquired about the potential effect of *APOL1* pathologic allelic variants (known as G1 and G2) on sphingolipid metabolism [8]. Transfection of human podocytes with wild-type *APOL1* induced profound changes in the sphingolipid profile, in particular a dramatic decrease in LacCer content, with no changes in gangliosides. Conversely, *APOL1* allele variants induced a significant increase in GD1α and sphingomyelin, and a decrease in GA1, ceramide, GlcCer, in addition to LacCer. No changes were observed in the most abundant gangliosides, such as GM3 and GM1. Strikingly, both variants and the WT form induced significant decreases in the enzymatic activities of hydrolases, such as α and β galactosidases, β-hexosaminidase, and glucosylceramidase, responsible for the catabolic reversed reactions on the same series. Conversely, when the authors studied these activities at the cell surface, they found the opposite effect, with an increased activity, especially when the G2 variant was overexpressed. The sphingolipid profile of lipid rafts was also changed, with significant decreases in gangliosides GD1α, GM3, GM1b, and GA1, along with Cer, GlcCer, and LacCer precursors, the changes varying depending on the APOL1 variant expressed. This suggests a role of APOL1, and possibly high density lipoproteins (HDL), in the regulation of ganglioside synthesis and degradation. In addition, changes induced in the sphingolipid profile by genetic variants G1 and G2 can contribute to podocyte alterations. However, most interestingly, changes in ganglioside species operate and are detectable especially at raft microdomains.

## 5. A Word by Ceramides

Ceramides are the central metabolites where pathways converge and diverge in the sphingolipid metabolic sphere. Therefore, the regulation of ceramide content has an impact on ganglioside synthesis and, conversely, the regulation of ganglioside synthesis and catabolism can have an effect on ceramide content. Ceramides themselves are bioactive lipids, but so are mostly their phosphorylated derivatives ceramide 1 phosphate (C1P) and sphingosine 1 phosphate (S1P). Ceramide levels are, therefore, tightly regulated in cells.

In a recent work, Li and Colls [51] characterized a mouse model in which the catalytic subunit of lysosomal acid ceramidase (Asah1) was invalidated specifically in podocytes. This enzyme is responsible for ceramide catabolism and is critical in cellular homeostasis. As expected, ceramides, in particular C16, accumulated in the glomeruli of these individuals. Interestingly, mice developed proteinuria in the absence of morphological changes in glomeruli following light microscopy observation. Conversely, electron microscopy revealed the foot process effacement. Altogether, these alterations are similar to those corresponding to MCNS. A double invalidation of *Asah1* and acid sphingomyelinase (*Smpd1*), an enzyme leading to ceramide production by sphingomyelin hydrolysis, corrected the ceramide levels and partially reversed the proteinuria and morphological changes observed in the single knockout. This points at ceramides as potential actors participating in the pathogenic mechanisms of podocytopathies. Moreover, the fact that these enzymes are lysosomal underlines the importance of these organelles in sphingolipid and glycosphingolipid homeostasis. No information is provided in the study on the status of the ganglioside flux.

In our work, we have found, along with a decrease in GM3, GM2, and GD3 in mouse podocytes transfected with human *CMIP*, an increased presence of LacCer, GlcCer, and several ceramides, consistent with a block in GM3 synthesis at the ST3Gal5/GM3 synthase level [7]. Our finding in mouse T-cells overexpressing human CMIP, consisting of a decrease in GM3 protein expression after 30 min of T-cell receptor (TCR) activation, seems to comfort the hypothesis of a GM3 synthesis blocking associated with CMIP overexpression. Interestingly, CMIP was first identified by subtractive cloning in T-cells [29]. Later, it was also detected in podocytes from MCNS patients [52], and the lesions induced in a mouse podocyte-specific transgenic model of CMIP overexpression are similar to those of the human MCNS [52]. Considering our results in view of the findings by Li and Colls [51], it is tempting to hypothesize that ceramides participate in CMIP-induced proteinuria due to their accumulation in podocytes, as a consequence of a GM3 synthesis blocking.

A nephropathy associated with ceramide accumulation is one of the features of Farber disease, a genetic disorder resulting from mutations in the *ASAH1* gene [53]. Ceramide levels are also increased in genetic steroid resistant nephrotic syndrome in association with mutated S1P lyase [54,55,56]. Likewise, pharmacological inhibition of this enzyme induces a nephrotic syndrome in rodents [57]. The nephropathy associated with diabetes also benefits from the targeting of ceramide accumulation by an adiponectin receptor agonist [58]. Acid sphingomyelinase overexpression also leads to ceramide accumulation and to glomerular sclerosis in mice [59]. All these reports point at ceramide accumulation as a contributor to podocyte and glomerular dysfunction, as a result of defects in different genes and proteins. Likewise, a block in ganglioside synthesis could represent an additional source of ceramide accumulation leading to similar consequences and place ceramides at a central point of podocyte injury.

However, the latter hypothesis is challenged by the seminal works of Fornoni et al. on the connection between sphingomyelinase-like phosphodiesterase 3b (SMPDL3b) and podocyte dysfunction. While SMPDL3b is overexpressed in diabetic nephropathy patients [60], recurrent FSGS patients present a decreased expression of this enzyme in podocytes [61]. This apparently contradicting scenario might indicate that these two diseases are too far apart pathogenically, though sharing the glomerular dysfunction. Nevertheless, to our knowledge, no systematic ceramide analysis has been performed to date on patients’ biopsies.

## 6. The Role of the Immune System

The direct implication of the immune system in podocytopathies such as IgA nephropathy, membranous nephropathy, and lupus nephritis is clear, as all these are based on the development of autoantibodies. In the case of INS, there are no immune deposits present in the glomerulus, yet an immune dysfunction at the origin of the pathology is supported by a consistent body of evidence [62]. Nonetheless, the precise pathogenic mechanisms involved in INS are far from being understood. Gangliosides exert capital functions in immune cells (for review [63,64]). They are present in both myeloid and lymphoid cell populations, as well as in hematopoietic stem cells [64]. Therefore, GM3 has been described in most immune cell types, except in eosinophils, basophils, and NK cells, in which GM1 and asialo GM1 have been reported and seem to represent the most abundant ganglioside species. O-acetylated forms of GD3 have also been described in T, B, and NK cells. An example of ganglioside function in the immune system is their implication in T-cell activation [65,66,67], where distinct profiles of gangliosides are characteristic of CD4 and CD8 cells. The ganglioside function in immune cells, as in podocytes, is related to their role in organizing membrane microdomains, but also to their interaction with cellular receptors and signal transduction. Consequently, a dysregulation of ganglioside metabolism can be expected to participate in the immune origin of INS. To date, an abnormal distribution of GM1 has been observed in T-cells overexpressing the INS marker CMIP, as well as a decreased GM3 synthase expression after TCR activation [7].

Activation of invariant NK cells by glycosphingolipid-1 (GSL-1), a bacterial monoglycosylceramide, is able to protect glomeruli and reverse the effects of adriamycin injection, an in vivo model of FSGS in the mouse [27]. Most interestingly, this protection was paralleled by increased expression of GM3 synthase in the kidney, concomitant with increased levels of Bcl-2, suggesting a protective role for GM3 in the kidney by engaging an antiapoptotic mechanism [68]. Although the link between adriamycin and GD3 synthase expression, and that between GSL-1 and GM3 expression are not well established, the same study suggests the involvement of TGF-β and SMAD signaling [27], and opens an interesting mechanistic field to understand the role of gangliosides in glomerular biology and in INS.

In systemic lupus erythematosus (SLE) patients, the presence of antibodies possessing sialidase activity [69] targeting gangliosides, and anti-ganglioside antibodies targeting asialo-GM1, GM1, GM2, GM3, GT1b, GD1b, and GD3 have been reported [70,71]. Increased GM1 has been observed in peripheral CD4+ T-cells [72]. Abnormal T-cell responses in SLE have been associated with an abnormal ganglioside profile [73,74]. Unfortunately, there is little information about the ganglioside profile in podocytes in SLE, which is limited to date to the increased presence of GM3 in the kidneys from a nephritic mouse model [41]. Other renal pathologies, such as IgA nephropathy, Henoch-Schönlein purpura nephritis, MCNS, mesangial proliferative glomerulonephritis, and membranoproliferative glomerulonephritis have been associated with the presence of antibodies against N-glycolyl GM3, a variant of GM3 characteristic of some cancer cells [75]. A case of hyperthyroidism accompanied by hematuria, proteinuria, proliferation of mesangial cells, and increased mesangial matrix with a focal segmental capillary wall abnormality, was attributed to the presence of a thyroid antibody targeting fucosyl-GM1. The latter was detected by immunofluorescence in the glomerular basement membrane [76]. The role of anti-ganglioside antibodies in the pathogenesis of these diseases is still unexplored.

## 7. A Call for Deep Analysis

All the observations described above, the subsequent hypotheses and insights, the potential pathogenic mechanisms, will benefit from further research based on state-of-the-art analytical strategies. It must be clarified that the reported gangliosides in the works published so far (i.e., GM3) do not correspond to single molecular entities, but to groups of molecules sharing a particular poly-sugar sialic acid-containing moiety, differing in the nature of the sphingoid base and fatty acid chains present, which complicates significantly the research from an analytical point of view.

Significant improvements have been made in the last 10 years in the analysis of gangliosides with the advent of lipidomics technologies [77]. Notably, the presence of polar oligosaccharide moieties that include sialic acid residues linked on their ceramide backbone, makes gangliosides particularly water-soluble. Therefore, during sample preparation, gangliosides tend to partition into a more polar or aqueous layer rather than in an organic layer, as observed with other lipid classes. Once extracted from their biological matrices, a low-cost procedure for the qualitative evaluation of the endogenous ganglioside pattern is represented by HPTLC. Using this technique, the gangliosides contained in the aqueous phase of the lipid extract are separated according to the different compositions of their carbohydrate structure using specific solvent systems. Lipids are then visualized using different strategies such as (i) chemical detection, (ii) binding assay using antibodies, (iii) carbohydrate recognition reagents, and identified by co-migration with the authentic lipid standard [78]. Despite the fact that this technique gives a rapid result related to the ganglioside composition of cells, the main issue is represented by the requirement of a relative high amount of biological sample. To increase the sensitivity of this methodology, the use of radioactive precursors of sphingolipids in the tracer concentration was exploited. In particular, cells are treated with [1-^3^H]-sphingosine or [3-^3^H]-sphingosine, or [^3^H]serine or [^14^C] serine to obtain metabolic labelling at the steady state of all cell sphingolipids. After incubation, gangliosides isolated in the aqueous phase are separated by TLC and radioactive lipids visualized by digital autoradiography. Exploiting the use of radioactivity and the sensitivity of digital autoradiography, the amount of biological samples to be analyzed is reduced by 1/100 with respect to that of the endogenous counterpart. As a weakness, the use of radioactive precursors is mainly applicable to cells in culture.

Interestingly, the use of [1-^3^H]-sphingosine gives also information related to sphingolipid turnover. [1-^3^H]-sphingosine, when administered to cells, is used for de-novo biosynthesis of sphingolipids, which become radioactive. Radioactive lipids are then degraded in lysosomes to obtain saccharides or choline, fatty acid, and [1-^3^H]-sphingosine. The radioactive sphingosine could be further used in the biosynthetic pathway or could be phosphorylated to obtain [1-^3^H]-sphingosine-1-phosphate (S1P). S1P is further degraded to hexadecenal and phosphoethanolamine, which is radioactive due to the presence of tritium on the first carbon of sphingosine. [^3^H]phosphoethanolamine is used for the biosynthesis of radioactive phosphatidylethanolamine. For this reason, the use of [1-^3^H]-sphingosine allows obtaining the metabolic labeling of all cell sphingolipids and also of phosphatidylethanolamine, which reflects the rate of sphingolipids and sphingosine turnover [8,78,79]. One of the main limitations of the HPTLC methodology is the incapability to provide quantitative data and the information related to ceramide structures, both evaluable by mass spectrometry (MS) analyses. MS, either electrospray ionization (ESI) or direct analysis ionization sources, such as matrix-assisted laser desorption ionization (MALDI), are routinely used to monitor and quantify the levels of gangliosides in biological samples [80]. Since different ganglioside species may have the same molecular mass, MS is often used in conjunction with separation techniques, such as chromatography and ion mobility, to separate isomers and characterize the complex and diverse chemical structures of gangliosides [81]. Recently, imaging MS has been used to facilitate ganglioside analysis in heterogeneous tissues, such as kidney and brain samples in particular, allowing to monitor their concentration and generate images of their molecular composition in fine anatomical structures and substructures [82].

## 8. Conclusions

In spite of the extreme structural diversity of gangliosides, only a handful of them have been identified in podocytes and their functions in the physiologic and pathologic conditions outlined (Table 1). Nevertheless, the cellular features of podocytes and the abundance of GM3 point at a relevance of gangliosides and their potential involvement in the pathophysiology of glomerulopathies. The role of the immune system in INS has been demonstrated, but the mechanisms are still in the dark.

As stated in Section 3.2, the general ST3Gal5 knockout mouse model does not develop any renal phenotype [83], whereas a podocyte-specific invalidation has not been developed to date. Cross breeding of this model with others can provide capital information on the role of ganglioside homeostasis in pathophysiology. One example in the context of kidney disease is the cross breeding of *ST3Gal5* knockouts with a transgenic mouse line bearing the juvenile cystic kidney mutation (jck), responsible for polycystic kidney. Breeding results in a milder polycystic pathology, suggesting that GM3 synthase is involved in the pathogenesis [84]. Similar strategies would complement analytical studies in developing consistent hypotheses on the pathophysiologic role of ganglioside synthases and gangliosides.

The implementation of MS-based lipidomic approaches, targeting the biochemical pathways for ganglioside biosynthesis and degradation, will allow a better understanding of the role played by these sphingolipids in kidney diseases. Clinical strategies, based on these state-of-the-art analytical approaches, might put this category of lipids as candidates for diagnostic and prognostic biomarkers of diverse forms of podocytopathies. A long path is still ahead.

## Figures and Tables

**Table 1:** Changes in the abundance of gangliosides and precursors associated with glomerular diseases.

Molecular Species	Pathology	Observed Changes	Reference
Ceramide	APOL1 associated FSGS	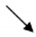 (podocytes)	[8]
	Proteinuria model (Asah1 KO mouse)	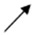 (lysosomes)	[51]
	Genetic steroid resistant nephrotic syndrome	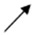 (glomeruli)	[54,55,56]
	Glomerular sclerosis (acid sphingomyelinase overexpression)	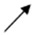 (glomeruli)	[59]
GlcCer	APOL1 associated FSGS	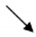 (podocytes)	[8]
LacCer	Lupus nephritis	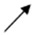 (kidney)	[40]
	APOL1 associated FSGS	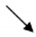 (podocytes)	[8]
GM3	INS (CMIP overexpression)	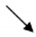 (podocytes)	[7]
	DN (streptozotocin rat model)	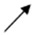 (kidney)	[35,36]
	DN (streptozotocin rat model)	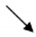 (glomeruli)	[37]
	DN (type 1 diabetes rat model)	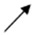 (glomeruli)	[38]
	DN (mouse model)	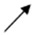 (glomeruli)	[39]
	APOL1 associated FSGS	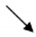 (podocyte rafts)	[8]
	Lupus nephritis (mouse model)	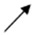 (kidney)	[41]
	IgA nephropathy, Henoch-Schönlein purpura nephritis, MCNS, mesangial proliferative glomerulonephritis, membranoproliferative glomerulonephritis	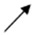 (antibodies)	[75]
GD3	PAN nephropathy rat model	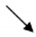 (kidney)	[26]
O-acetylated-GD3	Microalbuminuria associated with lead toxicity	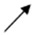 (kidney)	[25]
	PAN nephropathy rat model	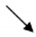 (kidney)	[26]
GM2	INS (CMIP overexpression)	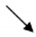 (podocytes)	[7]
	DN (glucosamine administration)	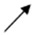 (mesangial cells)	[47]
GD2	INS (CMIP overexpression)	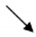 (podocytes)	[7]
GM1	DN (glucosamine administration)	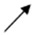 (mesangial cells)	[47]
	APOL1 associated FSGS	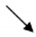 (podocyte rafts)	[8]
GD1α	APOL1 associated FSGS	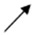 (podocytes)	[8]
	APOL1 associated FSGS	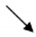 (podocyte rafts)	[8]
GA1	DN	Neg. correlation with eGFR	[48]
	APOL1 associated FSGS	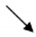 (podocytes)	[8]

## Abbreviations

APOL1	Apolipoprotein L1
Asah1	Lysosomal acid ceramidase
CMIP	lC-maf inducing protein
DN	Diabetes-associated nephropathy
EGF	Epidermal growth factor
eGFR	Estimated glomerular filtration rate
ESI	Electrospray ionization
FSGS	Focal and segmental glomerulosclerosis
Flt1	Vascular endothelial growth factor receptor 1
GalNAc	N-acetylgalactosamine
GlcCer	Glucosylceramide
GA	Asialo gangliosides
GD	Di-sialo gangliosides
GM	Mono-syalo gangliosides
GP	Penta-sialo gangliosides
GQ	Quadri-sialo gangliosides
GSL-1	Glycosphingolipid-1
GT	Tri-sialo gangliosides
HDL	High density lipoproteins
HPTLC	High performance thin layer chromatography
INS	Idiopathic nephrotic syndrome
LacCer	Lactosylceramide
MALDI	Matrix assisted laser desorption ionization
MCNS	Minimal change nephotic syndrome
NEU	Neuraminidase
PAN	Puromycin aminonucleoside nephropathy
ST8Sia1	Ganglioside D3 synthase
SMPDL3b	Sphingomyelinase-like phosphodiesterase 3b
ST3Gal1	alpha-2,3-sialyltransferase
ST3Gal5	Ganglioside M3 synthase
SLE	systemic lupus erythematosus
TCR	T-cell receptor
VEGF	Vascular endothelial growth factor
